# Trends of burning mouth syndrome: a bibliometric study

**DOI:** 10.3389/fneur.2024.1443817

**Published:** 2024-07-23

**Authors:** Xuanyu Lin, Ruihui Jin, Wanyu Huang, Yicai Ye, Jing Jin, Wenzong Zhu

**Affiliations:** ^1^Wenzhou Hospital of Integrated Traditional Chinese and Western Medicine of Zhejiang Chinese Medical University, Wenzhou, China; ^2^Liaoning University of Traditional Chinese Medicine, Shenyang, China

**Keywords:** research trends, burning mouth syndrome, CiteSpace, bibliometric analysis, visualization

## Abstract

**Objectives:**

This study utilizes bibliometric analysis to map the current research landscape and forecast emerging trends within the domain of Burning Mouth Syndrome (BMS).

**Materials and methods:**

A comprehensive review of literature related to BMS was conducted, drawing from the Web of Science Core Collection (WoSCC) from 2008 to 2023. The analysis included both publication types “Article” and “Review Article.” Advanced quantitative techniques and visual analytics tools, including CiteSpace, VOSviewer, Tableau, and the Map Equation online platform were utilized to analyze the academic publications within this domain.

**Results:**

Our analysis incorporated 497 articles on BMS. The data exhibit a progressive increase in the annual volume of publications from 2008 to 2023. In terms of geographic and institutional contributions, the United States of America (with 80 publications) and Nihon University (with 26 publications) emerged as leading entities in BMS research, while the Netherlands and England were identified as central to international collaboration efforts. Prominent researchers in this field include Adamo Daniela (18 publications) and Sun Andy (16 publications). Furthermore, the most cited works were authored by Jääskeläinen SK. An examination of the journals in which these articles were published showed a dominance of dental journals, highlighting significant interest and research efforts in BMS within the dental research community.

**Conclusion:**

The steady growth in BMS research signifies the formation of a robust core of researchers and demonstrates the maturation of the field. Despite this progress, the findings highlight a notable deficiency in cross-institutional and cross-regional collaborative efforts. Keyword cluster analysis has revealed “management” as a persistently relevant theme, with “pain modulation” emerging as the current focal interest. Additionally, “blood profile,” “pernicious anemia,” and “folate” have been identified as prospective areas of growing interest, suggesting important directions for future investigations.

**Clinical relevance:**

This bibliometric analysis reveals the research landscape of BMS, aiming to highlight potential collaborative opportunities and define future research directions. These insights are invaluable for guiding subsequent investigations and carving new paths in the exploration of BMS.

## 1 Introduction

Burning Mouth Syndrome (BMS) is recognized as one of the predominant conditions affecting the oral mucosa, with a reported incidence rate of 1.73% in the general population and 7.72% among clinical patients (1). Predominantly affecting middle-aged and elderly women, BMS often has a negative impact on patients’ lives (2). Patients commonly report symptoms such as a burning sensation and pain in the tongue and oral mucosa, accompanied by numbness, taste disturbances, and psychological effects including anxiety, depression (3, 4), cognitive dysfunction (5), and other symptoms with no obvious organic lesions or histopathological changes found upon clinical examination (6). The terminology and diagnostic frameworks for BMS are subjects of ongoing scholarly debate. A notable Delphi study advocated for refining BMS as “Burning Mouth Disorder” (BMD) and suggested the exclusion of “emotional” factors from its diagnostic criteria (7). Current etiological hypotheses involve central nervous system changes, oral microbial dysbiosis, and psychosocial factors (8–11), thereby necessitating interdisciplinary research approaches that bridge oral, neurological, and endocrine studies (12).

Clinically, the diagnosis of BMS is predominantly based on the subjective description of abnormal sensations in the patient’s tongue or other parts of the oral cavity, often leading to a diagnosis by exclusion (7). The absence of a universally accepted classification system for BMS has prompted researchers to propose various frameworks, ranging from distinctions based on systemic versus neurological factors and local versus psychological factors (13), to classification based on causative factors, proposing five different subtypes (14). Despite the availability of treatments ranging from nutritional nerve medications, low-energy laser therapy to antipsychotic drug therapy, their effectiveness remains unclear (15, 16).

Current research on BMS has focused on diagnostic criteria (7), etiological factors (9), and other pertinent discussions, indicating a mature field. However, there is an observed deficiency in quantitative and qualitative analysis. Therefore, conducting a bibliometric analysis is imperative to elucidate the foundational structure and emerging focal points of BMS research. Bibliometric research employs statistical techniques to review the body of literature within academic disciplines, offering insights into important authors, institutions, and countries, thereby facilitating a comprehensive understanding of the field (17). This study utilizes tools such as CiteSpace, VOSviewer (18), Tableau, and the Map Equation to conduct an in-depth analysis of the BMS literature, aiming to map the chronological distribution of publications, identify research hotspots, assess journals contributions, and visualize prospective research trajectories.

## 2 Methods

### 2.1 Data sources and retrieval strategies

This study sourced literature from the Web of Science Core Collection (WoSCC), utilizing the search formula TS = (“BMS” OR “burning mouth syndrome” OR “buring mouth syndrome” OR “burn the mouth syndrome”). The search, completed on January 21, 2024, spanned literature from 2008 to 2023, exclusively in English, including both “Article” and “Review Article” types.

### 2.2 Data handling

Initial searches retrieved9,361 articles, which were manually screened by year and exported in “Plain text file” format for analysis. A total of 497 records met the criteria. Analytical tools used included CiteSpace (v6.2.R3) for country analysis and co-citation network mapping, and VOSviewer 1.6.20 for journal publication and citation count analyses. Tableau software and the Map Equation online platform facilitated the visualization of national and co-citation analysis of literature.

### 2.3 Software parameter settings

For the visualization analyses, we employed CiteSpace v6.2.R3, VOSviewer 1.6.20, Tableau software, and the Map Equation online platform. In CiteSpace, “Time Slicing” was set to one year. No “Pruning” was applied for mapping author publishing trends, institutions, and journal analyses. “Pathfinder” and “Pruning” sliced networks were activated for analyses involving authors, country distributions, co-cited literature, and keyword The G-index’s K value was set to 25, with the Top N% was set to 10% for analyses of author trends, institutions, journals, and country distributions. For co-cited literature, keyword clustering, and timeline analyses, the G-index’s K value was set to 20, with the Top N% also set to 10%. Adjustments to the nodes and connections in the visual graphs were made according to the analysis focus to optimize outcomes. Default settings were applied to VOSviewer, Tableau, and the Map Equation online platform.

## 3 Results and analysis

### 3.1 Analysis of publication outputs

**Figure 1:**
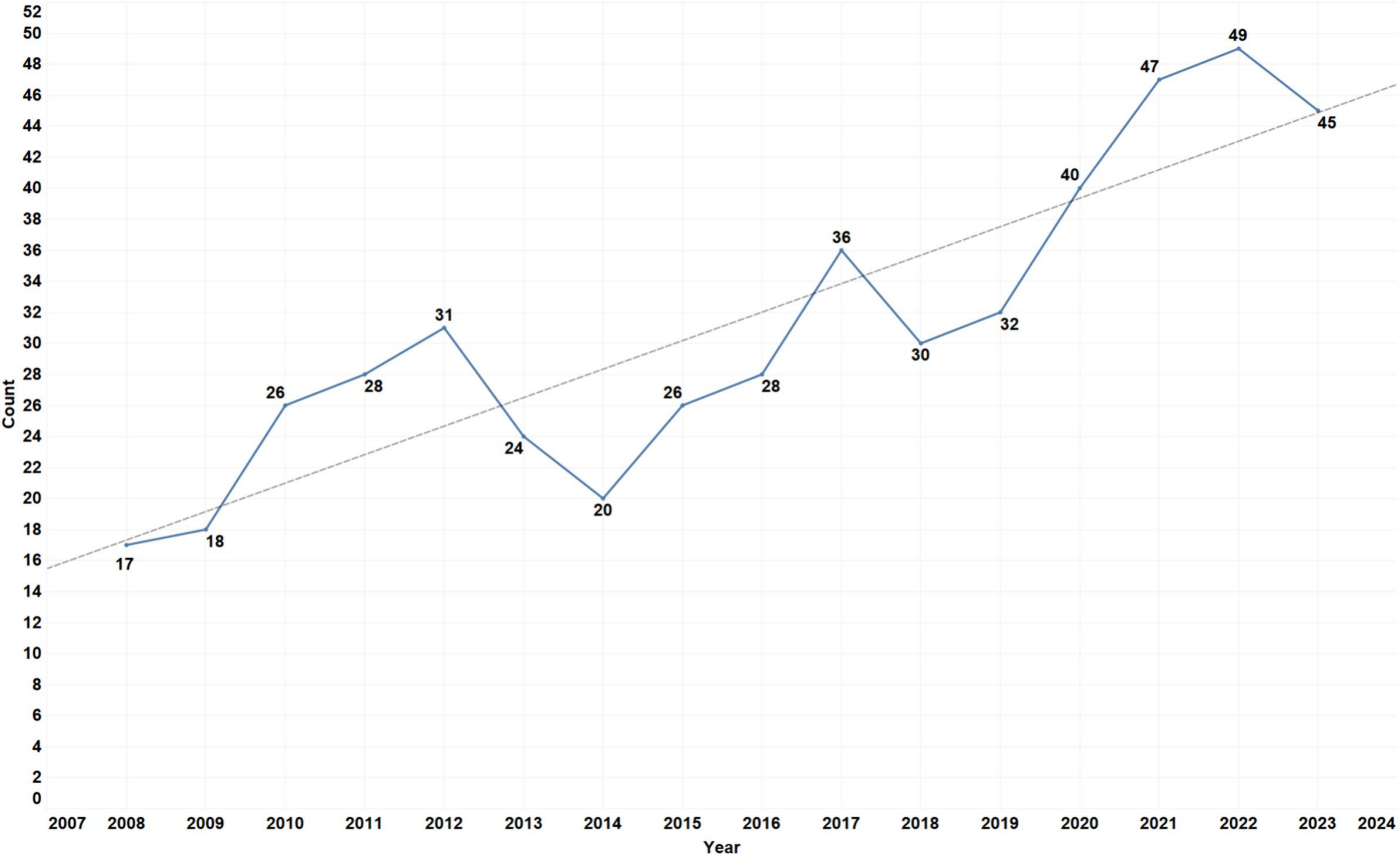
Annual distribution of peer-reviewed papers on BMS.

Within the WoSCC, we initially identified 9,361 articles related to BMS. Out of these, 498 articles met the inclusion criteria for our study. Figure 1 demonstrates the temporal distribution of published articles across 16 consecutive years. The vertical axis quantifies the publications *per annum*, while the horizontal axis corresponds to the timeline of these publications. A gradual upward trend in research output related to BMS is observed, despite minor fluctuations during the periods of 2013–2016 and 2018–2019. Notably, in 2020, despite the global disruptions caused by the COVID-19 pandemic, there was a significant increase in publications related to BMS, with the total exceeding 40 articles. This surge may indicate a potential link between the rising incidence of BMS and the prevalence of the novel coronavirus. In 2022, there was a resurgence in research interest within this domain. By the end of 2023, the WoSCC recorded 45 publications pertinent to BMS, marking the highest annual output since 2020 and reflecting an escalating academic recognition of the significance of this field.

### 3.2 Analysis of national and regional contributions

The analyzed articles were sourced from 48 distinct countries, with the leading quartet of contributors responsible for more than 50% of the total scholarly output. The United States emerged as the forefront of BMS research with 80 publications (16.0%), closely followed by Italy with 71 publications (14.2%), and Japan with 56 publications (11.2%), as detailed in Table 1. This distribution not only highlights the global nature of BMS research but also showcases the extensive geographic diversity contributing to the field. The analysis of the geographical spread of these contributions provides insights into the focal areas of research and expertise, emphasizing the significant role of international collaboration in enhancing the collective understanding and discourse in BMS research.

**Table 1:** The top 10 nations by publication count and centrality.

Rank	Count	Centrality	Year	Country
1	80	0.2	2008	United States
2	71	0.37	2008	Italy
3	56	0.07	2008	Japan
4	46	0	2008	Brazil
5	45	0.07	2008	Spain
6	32	0	2009	South Korea
7	32	0.85	2008	England
8	29	0.2	2008	France
9	27	0.27	2009	China
10	21	0	2011	Taiwan, China

**Figure 2:**
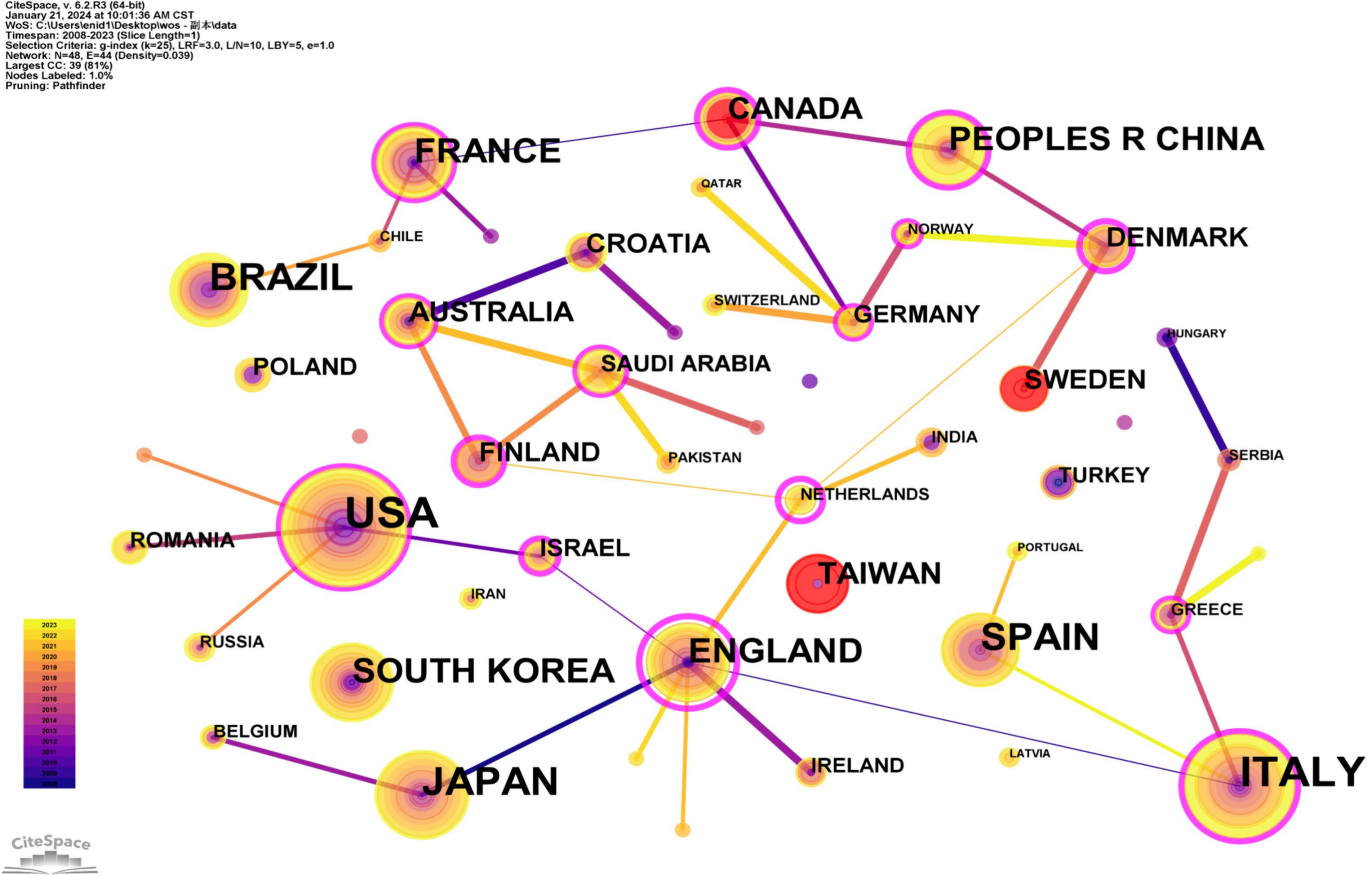
Network of international collaborations among countries/regions.

**Figure 3:**
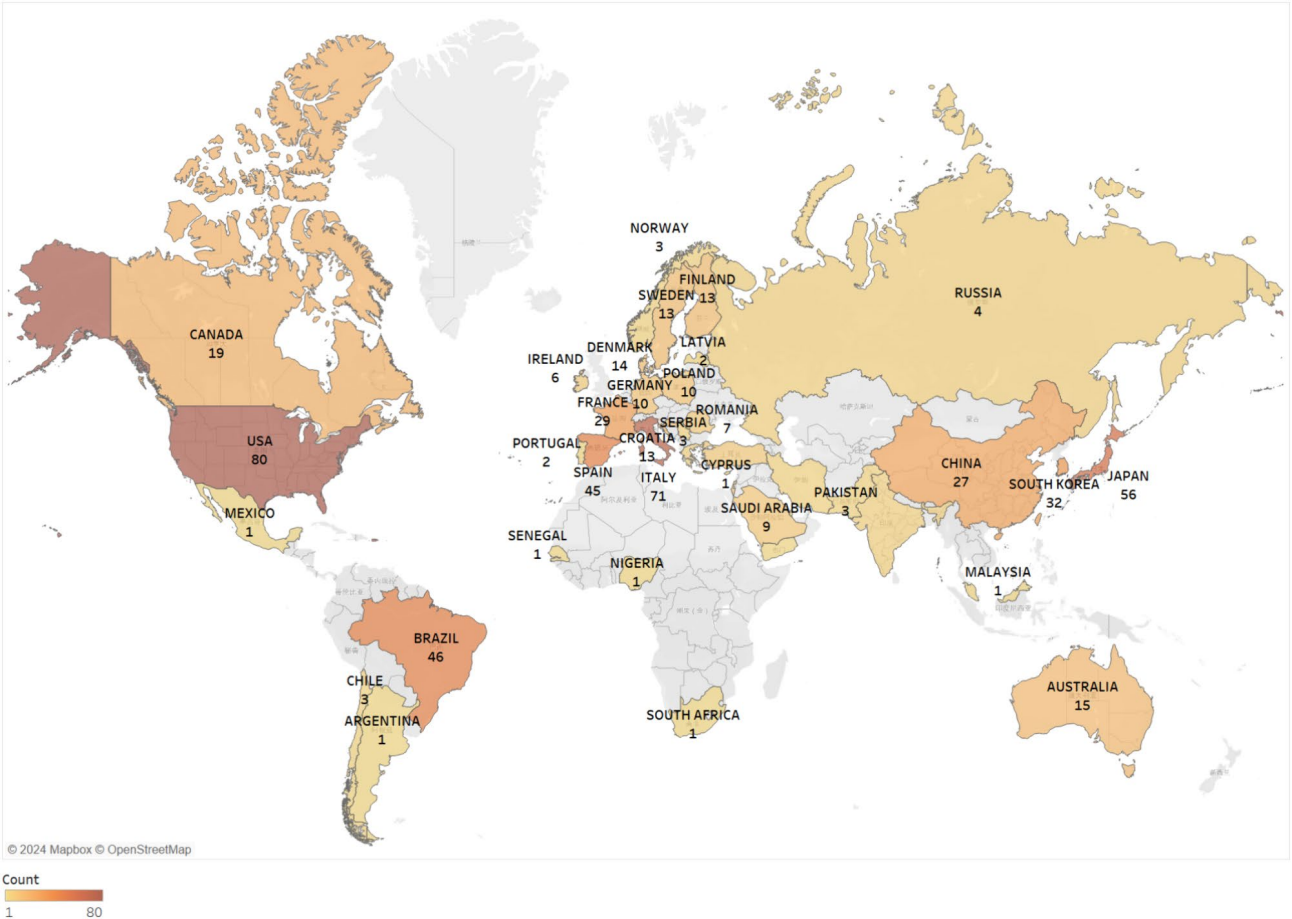
Map illustrating international collaborations among countries/regions.

Utilizing CiteSpace and the Map Equation online platform for visual analytics (Figures 2, 3), we identified key nations such as the Netherlands (centrality score of 0.86) and England (centrality score of 0.85) as central figures in the landscape of international collaboration, despite their relatively modest publication outputs. When employing the “Show/Hide Citation/Frequency Burst” mode in CiteSpace, these nations are denoted by read nodes, indicating a swift and significant increase of BMS-related scholarly work within a short period. Additionally, this analysis revealed that Canada, Sweden, and Taiwan (China), have experienced a surge in publications, suggesting an increasing local interest in BMS research.

### 3.3 Analysis of institution

**Figure 4:**
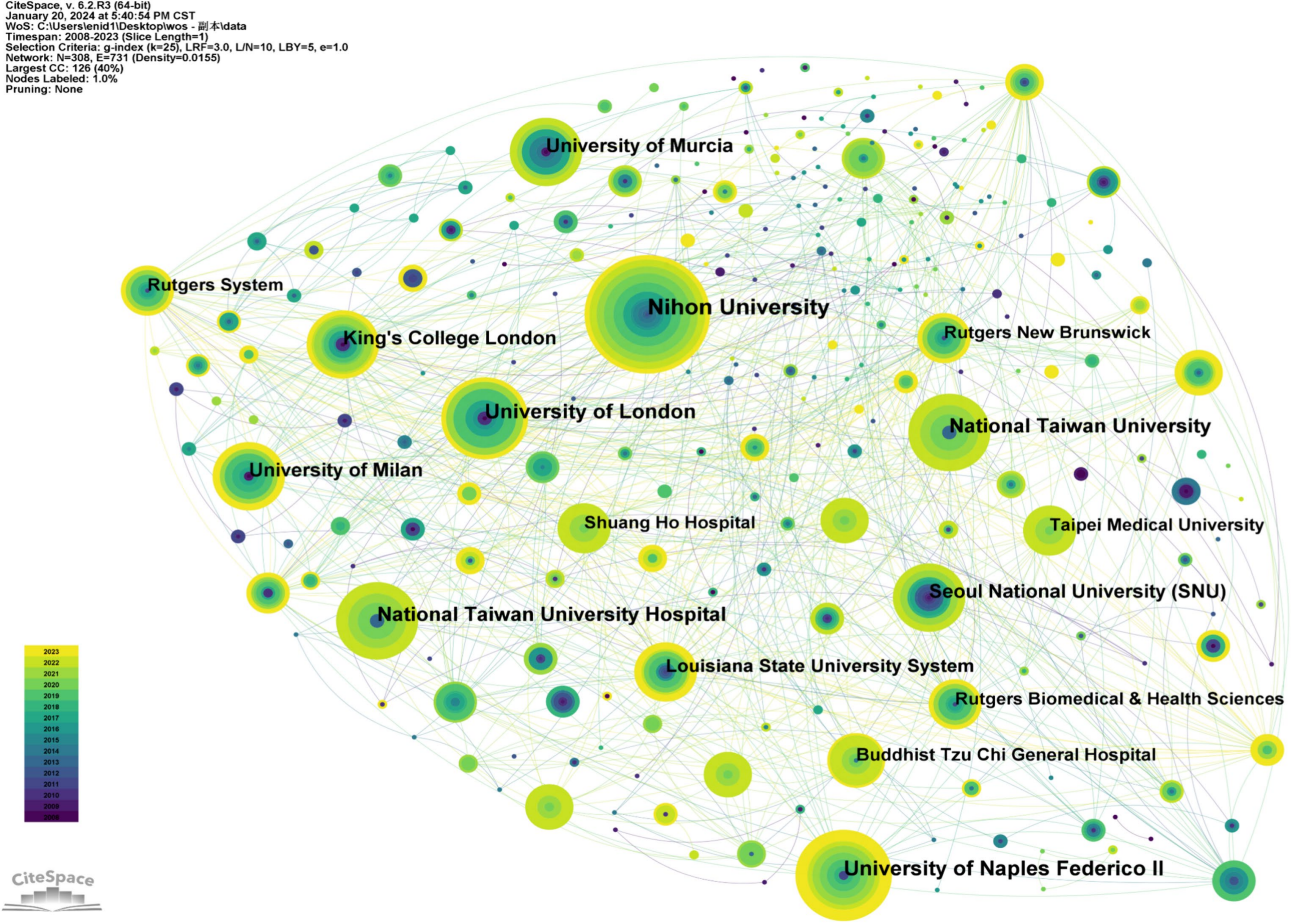
Network of institutional collaborations.

A total of 306 institutions have significantly contributed to the advancement of research on BMS, with Table 2 listing the top 10 institutions in terms of research output. Leading this list is Nihon University, with26 publications (5.2%), followed by the University of Naples Federico II with 20 publications (4.0%), and the University of London with 18 publications (3.6%). Notably, the list includes two institutions each from Taiwan and England, indicating a global spread of research efforts. Using CiteSpace, a graphical representation was created to map the distribution of research institutions within the field of BMS (Figure 4). In this graphical analysis, institutional collaborations are depicted through the interconnections between data nodes. The thickness of these links indicates the strength and relevance of collaborative efforts, offering insights into the collaborative networks and synergy among institutions in BMS research. Such visual analyses are important in highlighting the interconnectedness and collaborative dynamics within the research community, thereby enhancing the identification of potential partnerships and promoting the collective progression of BMS research. Specifically, Nihon University has emerged as an important institution, with its diverse research spanning neurology, endocrinology, psychology, and epidemiology, enriching the body of knowledge on BMS (1, 12, 19).

**Table 2:** The top 10 institutions in BMS research, 2008–2023.

Rank	Count	Centrality	Institutions	Country
1	26	0.07	Nihon University	Japan
2	20	0.02	University of Naples Federico II	Italy
3	18	0.03	University of London	England
4	17	0	National Taiwan University Hospital	Taiwan, China
5	17	0	National Taiwan University	Taiwan, China
6	15	0.07	University of Milan	Italy
7	15	0	University of Murcia	Spain
8	15	0	Seoul National University (SNU)	South Korea
9	15	0.03	King’s College London	England
10	13	0.05	Louisiana State University System	United States

### 3.4 Analysis of journals

An analysis performed with VOSviewer identified 180 journals that have published articles related to BMS from 2008 to 2023, covering a wide range of academic disciplines such as dentistry, neurology, and pain science. This diversity indicates the multidisciplinary interest in BMS, with significant contributions that enrich understanding, diagnosis, and treatment approaches for BMS from a scholarly perspective. Table 3 presents the top ten journals with the highest number of publications on BMS, led by *Oral Diseases,* followed by the *Journal of Oral Pathology & Medicine*, and the *Journal of Oral Rehabilitation*. Notably, *the Journal of Oral Pathology & Medicine* has the highest citation count (940 citations), indicating its important role in the academic discussion on BMS. These journals predominantly fall under the category of Dentistry, Oral Surgery & Medicine, highlighting the centrality of dental research in the BMS discourse. *Oral Diseases* is distinguished not only by its publication volume but also by its impact fact (IF 3.8), reflecting its influence in the field.

**Table 3:** The top 10 most influential journals in BMS research, 2008–2023.

Rank	Full journal title	Number of articles	Total citations	IF2023	WOS Categories
1	Oral Diseases	39	787	3.8	Dentistry, Oral Surgery & Medicine
2	Journal of Oral Pathology & Medicine	30	940	3.3	Dentistry, Oral Surgery & Medicine
3	Journal of Oral Rehabilitation	21	408	2.9	Dentistry, Oral Surgery & Medicine
4	Journal of Dental Sciences	18	137	3.5	Dentistry, Oral Surgery & Medicine
5	Medicina Oral Patologia Oral Y Cirugia Bucal	17	428	2.2	Dentistry, Oral Surgery & Medicine
6	Journal of Oral & Facial Pain and Headache	17	155	2.5	Dentistry, Oral Surgery & Medicine
7	Clinical Oral Investigations	14	169	3.4	Dentistry, Oral Surgery & Medicine
8	Oral Surgery Oral Medicine Oral Pathology Oral Radiology	13	143	2.9	Dentistry, Oral Surgery & Medicine
9	Journal of Oral Science	9	114	1.9	Dentistry, Oral Surgery & Medicine
10	Journal of Orofacial Pain	8	300	2.8 (2015)	No

**Figure 5:**
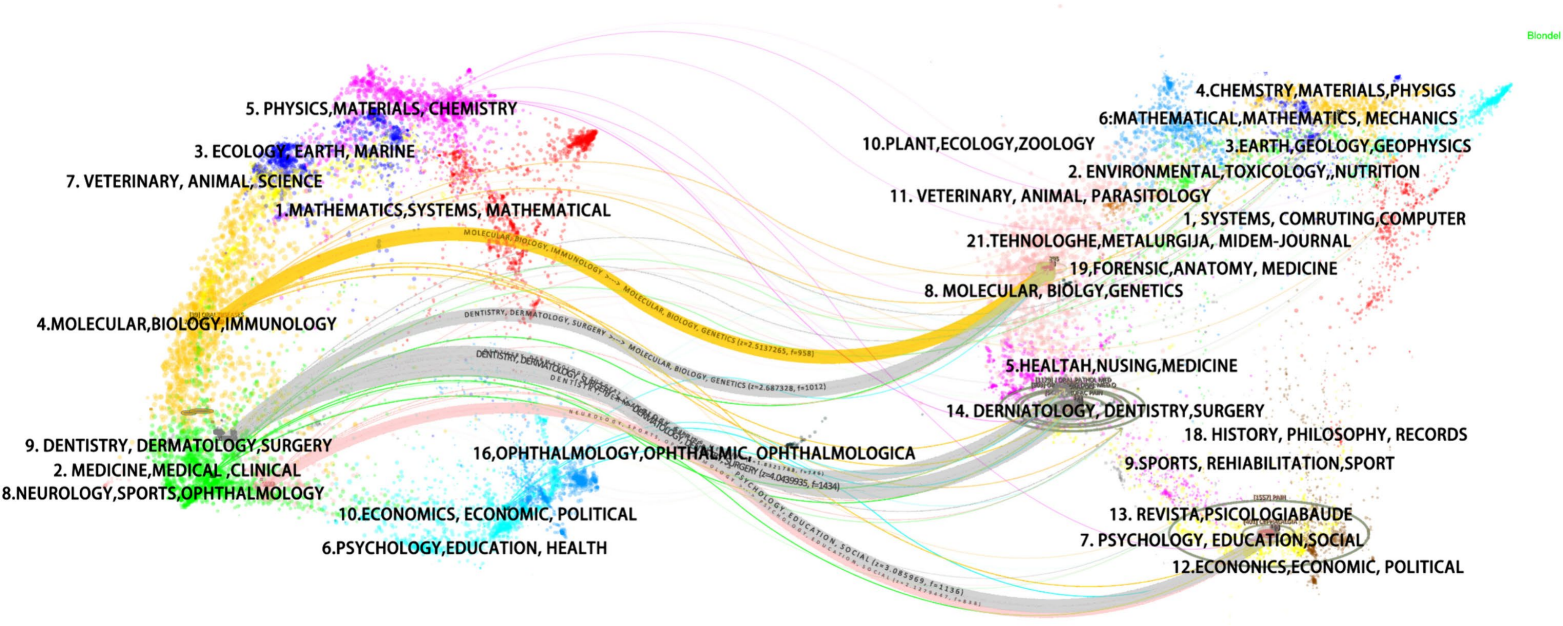
Dual-map overlay of BMS research.

Further, a dual-overlay map was constructed (Figure 5) to elucidate the citation dynamics within BMS research. The “dual-map overlap” technique showcases the distribution of citing journals (left) against the backdrop of cited journals (right), with curved lines mapping the citation flows between them (20). This mapping reveals that BMS-related citations predominantly engage journals within Dentistry, Dermatology, and Surgery, with notable intersections with Psychology, Education, and Social Sciences, illustrating the interdisciplinary nature of BMS research.

### 3.5 Analysis of author contributions in BMS

**Figure 6:**
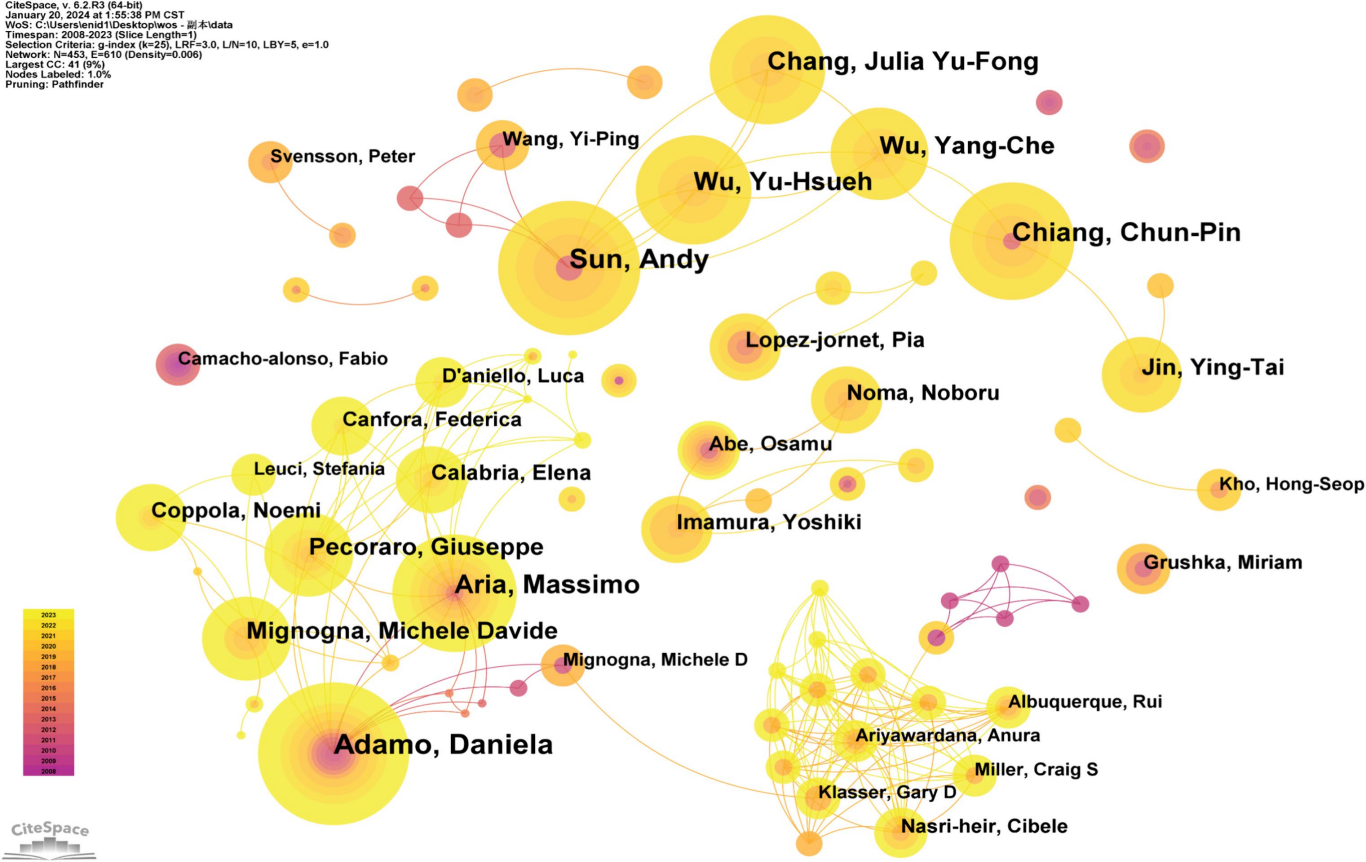
Author collaboration network in BMS research.

Within the domain of BMS research, a remarkable number of 414 authors has substantially contributed to the field’s development. These authors have been crucial in advancing the understanding of BMS through their extensive research, insights, and expertise. Collectively, their endeavors have propelled the expansion of knowledge and facilitated progress in this field. Table 4 lists the top 10 most prolific authors, with Adamo Daniela leading the count, having authored 18 articles, followed by Sun Andy with 16 publications, and Aria Massimo contributing 15. Utilizing CiteSpace software, author nodes were analyzed to construct a map showcasing the collaborative network among these researchers, with Figure 6 depicting the main collaborative relationships.

**Table 4:** The top 10 leading authors in BMS, 2008–2023.

Rank	Number of articles	Authors
1	18	Adamo Daniela
2	16	Sun Andy
3	15	Aria Massimo
4	15	Mignogna Michele Davide
5	14	Chiang Chun-Pin
6	13	Chang Julia Yu-Fong
7	13	Wu Yu-Hsueh
8	11	Wu Yang-Che
9	10	Pecoraro Giuseppe
10	9	Jin Ying-Tai

These core researchers have established themselves as the backbone of academic research in BMS, consistently producing a plethora of high-quality research that catalyzes further investigation within the field. According to Price’s Law (21), the criteria for core authorship in a research domain are derived from the formula *n* = 0.749√Nmax, where Nmax represents the highest number of publications by an individual author. Based on this criterion, and considering data from 2008 to 2023, an author must have a minimum of three publications to qualify as a core contributor in BMS research. This analysis identified 77 core authors, who collectively have contributed 424 articles, comprising 85.3% of the total publication output in this domain. These results align with Lotka’s Law (22), indicating the formation of a robust, central cohort of authors within the BMS research field.

### 3.6 Analysis of co-cited literature in BMS research

Table 5 provides a comprehensive list of the top ten publications ranked by co-citation strength, a metric indicating the frequency at which two articles are simultaneously cited by subsequent research. Co-citation analysis, a concept pioneered by the U.S. intelligence community in 1973, is important in delineating the intellectual framework and thematic relationships within a given field. Henry Small, a prominent researcher, articulated co-citation analysis as the simultaneous citation of two articles by a third, thereby highlighting the interconnectedness and relevance of scholarly works within a particularly domain (23).

**Table 5:** The top 10 co-cited references, 2008–2023.

Rank	Title	Authors	Publication year	Co-cited strength	Total citation
1	Pathophysiology of primary burning mouth syndrome	Jääskeläinen SK	2012	57	174
2	Burning mouth syndrome	Jääskeläinen SK	2017	47	105
3	Role of psychological factors in burning mouth syndrome: a systematic review and meta-analysis	Galli F	2017	46	92
4	International Classification of Orofacial Pain, 1st edition (ICOP)	Benoliel R	2020	46	291
5	Headache Classification Committee of the International Headache Society (IHS) The International Classification of Headache Disorders,3rd edition	Olesen J	2018	45	4,252
6	Is burning mouth syndrome a neuropathic pain condition?	Jääskeläinen SK	2018	32	59
7	Burning mouth syndrome: a systematic review of treatments	Liu YF	2018	32	65
8	Effect of lingual nerve block on burning mouth syndrome (stomatodynia): a randomized crossover trial	Grémeau-Richard C	2010	29	110
9	Burning mouth syndrome: update	López-Jornet P	2010	28	113
10	The association between burning mouth syndrome and sleep disturbance: a case–control multicentre study	Adamo D	2018	28	44

**Figure 7:**
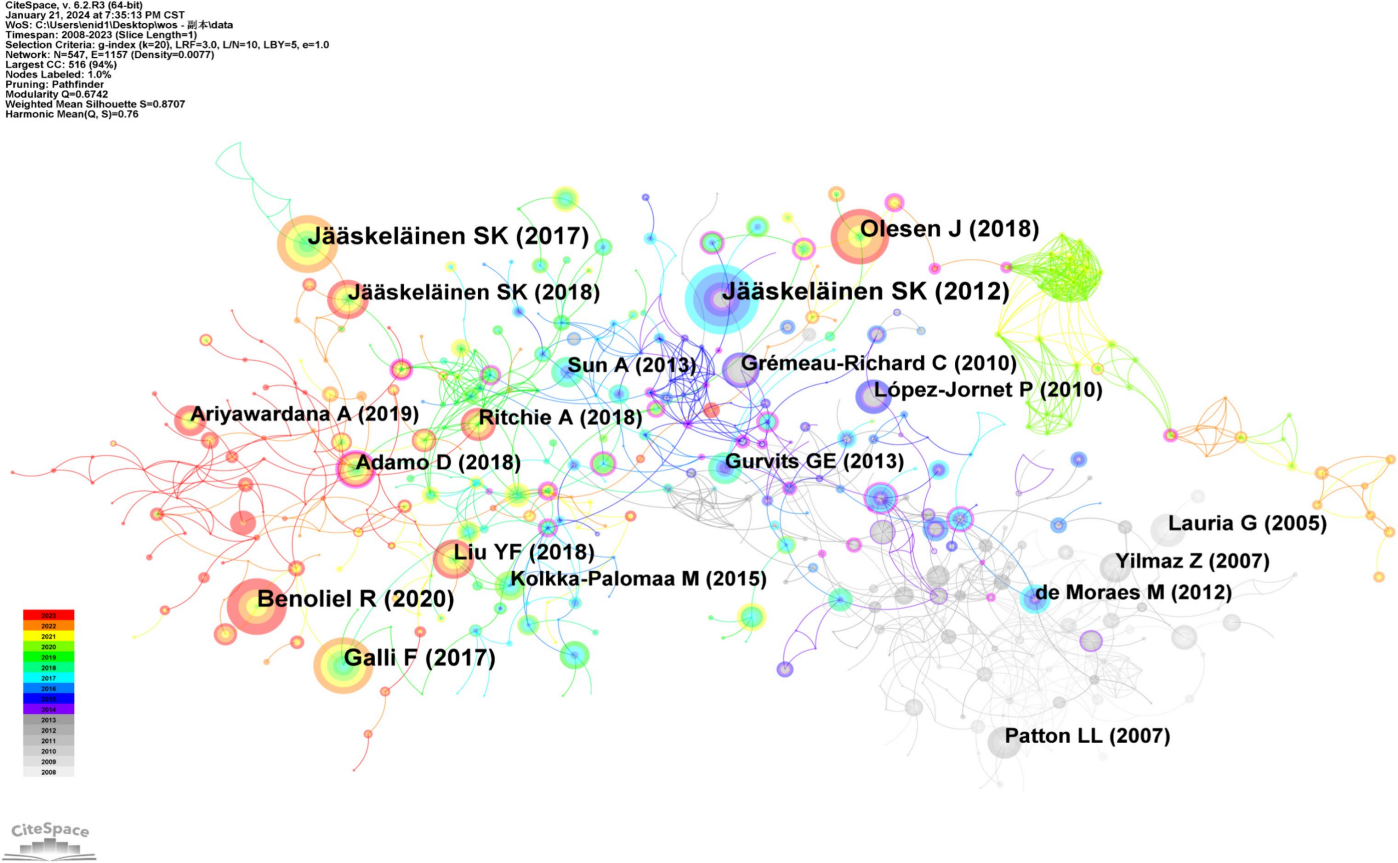
Network of co-cited references.

Utilizing CiteSpace for co-citation analysis (Figure 7), it was determined that “Pathophysiology of primary burning mouth syndrome” by Jääskeläinen SK, published in *Clinical Neurophysiology* in 2012, holds the highest co-citation strength with 174 citations. This article, originating from the University of Turku, advocates for the clinical diagnosis of primary BMS to include at least three subclinical neuropathic pain states, necessitating varied treatment strategies tailored to the specific neural mechanisms involved (24). Furthermore, “Burning mouth syndrome” by Jääskeläinen SK, a thorough review published in *Cephalalgia* in 2017, is the second most-co-cited article. This review comprehensively addresses the clinical characteristics, pathophysiological mechanisms, and broader aspects of BMS (25).

**Figure 8:**
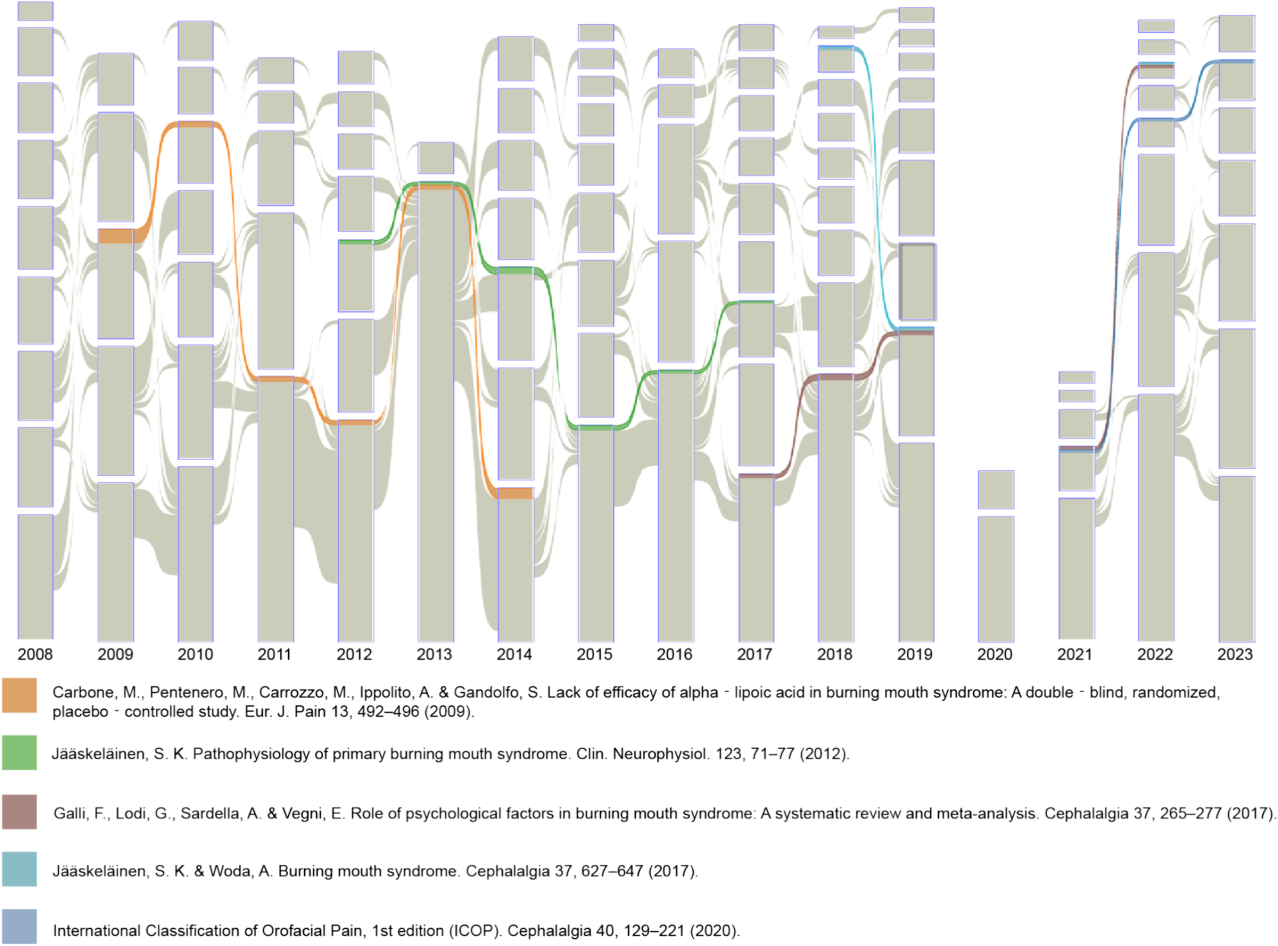
Alluvial flow visualization of co-cited references.

A Sankey diagram was also developed (Figure 8) to map the citation flow of significant literature in BMS research over the past sixteen years. The diagram incorporates color-coded segments that highlight the literature consistently cited across consecutive years. Such citation patterns suggest these works sustained scholarly recognition and their ongoing significance and relevance in the field. This diagram includes five articles, notably Jääskeläinen (24), Jääskeläinen and Woda (25), Galli et al. (26), Benoliel (6), and Carbone et al. (27). While Carbone et al. (27) displayed a notable alluvial flow, its co-citation strength was observed to be 21. This article presents a controlled study on the effectiveness of Alpha-Lipoic Acid (ALA) in BMS treatment, concluding a negligible effect on patients (27). A temporal citation analysis revealed that Carbone M’s study in 2009 ceased to attract citation since 2015. This indicates its role as a classic article in the nascent phase of BMS research. It also suggests a shift in research focus, with researchers increasingly investigating new treatment paradigms for BMS.

### 3.7 Analysis of keywords in BMS research

**Figure 9:**
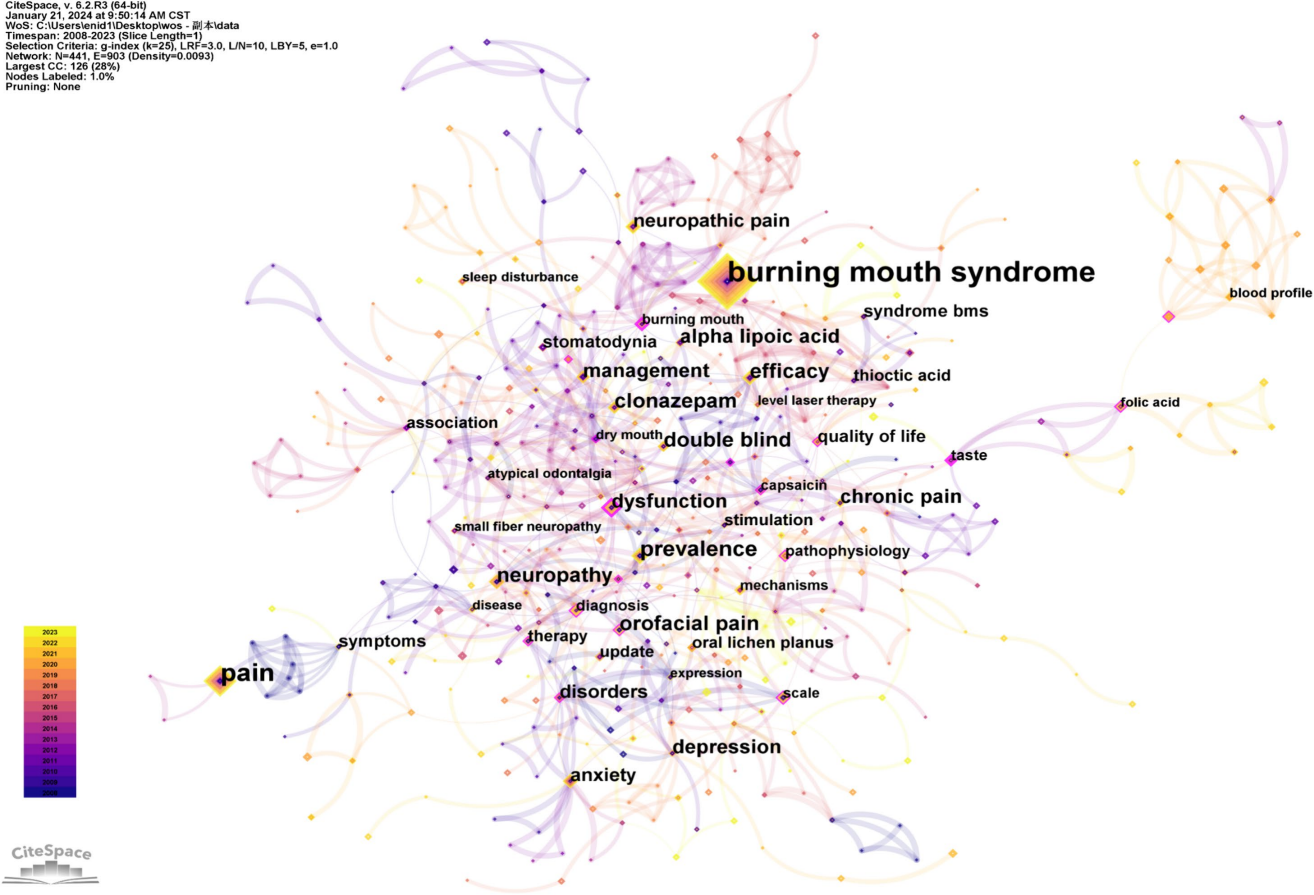
Map of keywords in BMS research.

**Figure 10:**
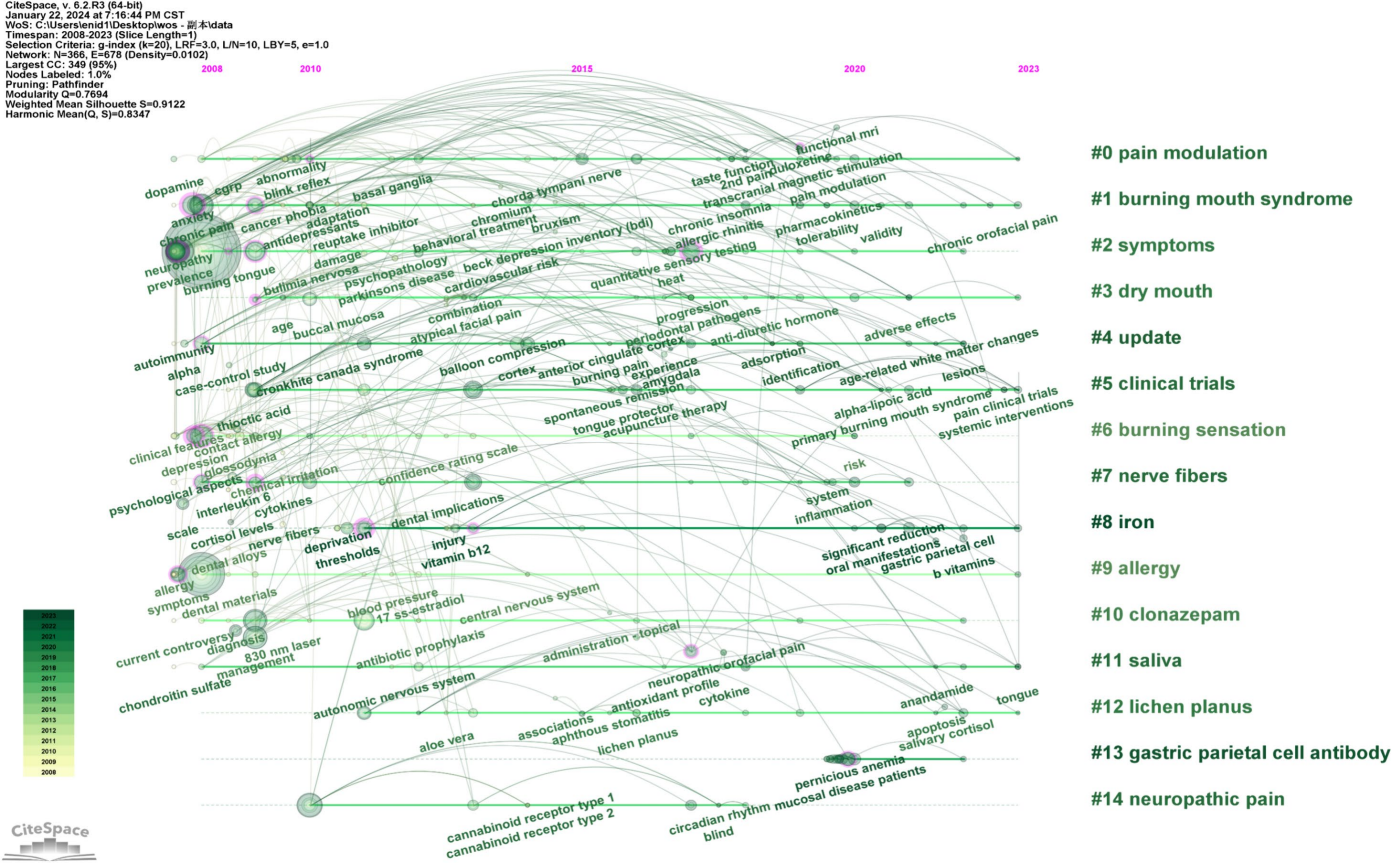
Timeline view of keyword clustering analysis.

**Figure 11:**
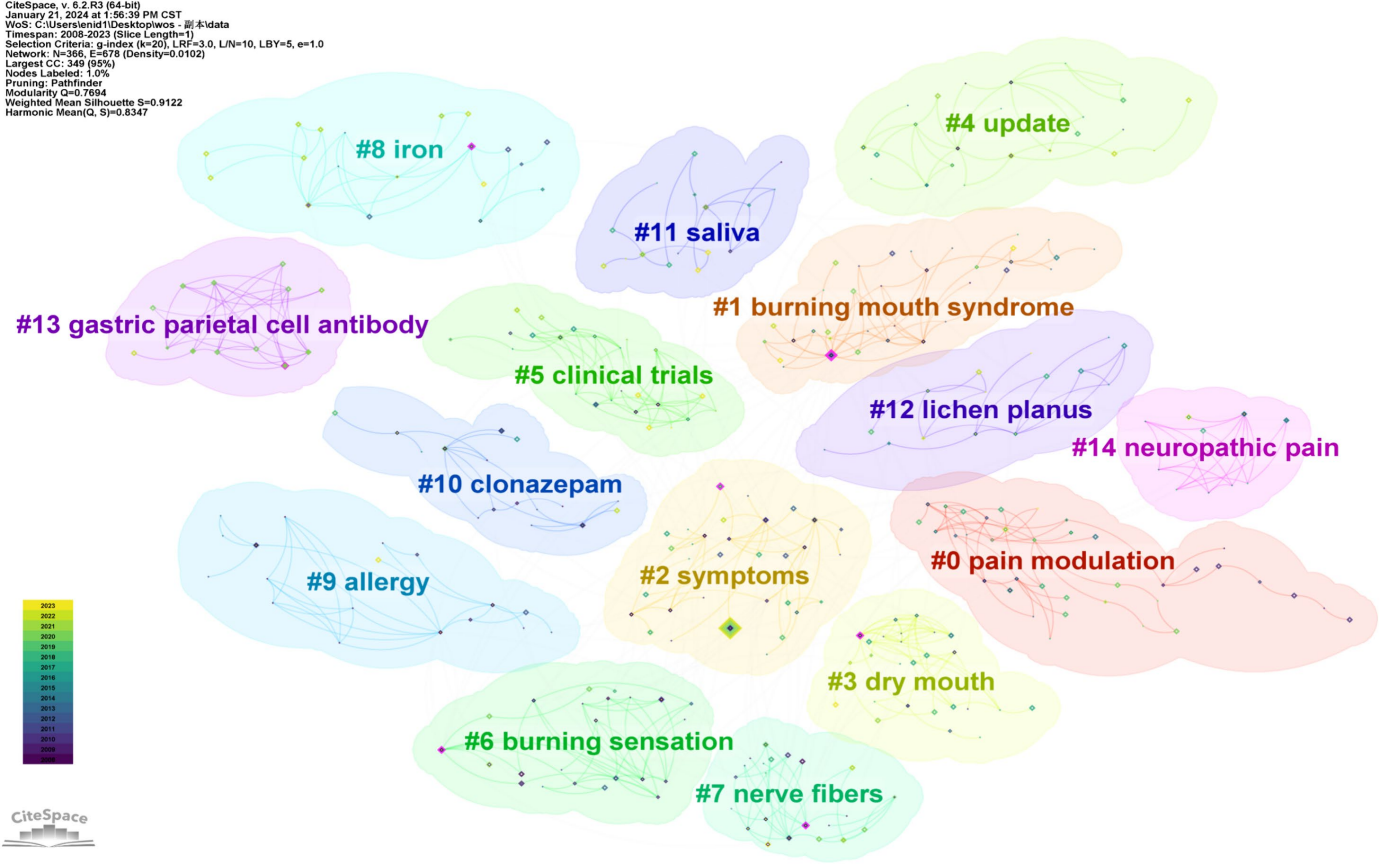
Cluster view of keyword clustering analysis.

**Figure 12:**
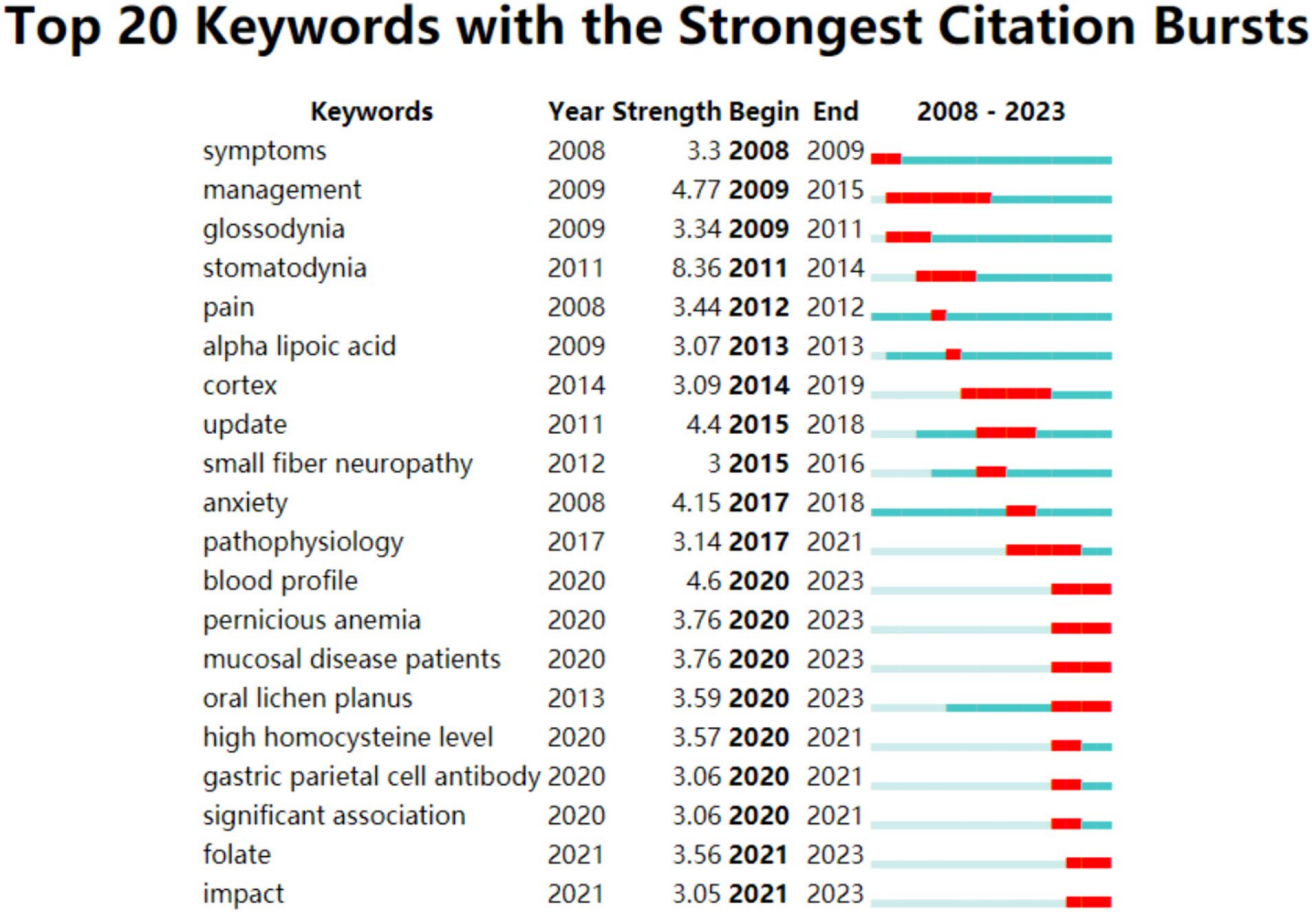
Top 25 keywords with major citation bursts.

A comprehensive keyword analysis was conducted using CiteSpace, as illustrated in Figures 9–12. Table 6 presents the top 10 keywords by frequency from 2008 to 2023, with “Burning mouth syndrome” leading at 358, followed by “Pain” (144) and “Prevalence” (71). The application of the Log-Likelihood Ratios (LLR) algorithm in CiteSpace facilitated the generation of a timeline chart, which categorizes keywords into distinct clusters, revealing significant thematic areas in BMS research (Figure 10).

**Table 6:** The top 10 keywords in BMS research, 2008–2023.

Rank	Frequency	Keywords
1	358	Burning mouth syndrome
2	144	Pain
3	71	Prevalence
4	58	Efficacy
5	53	Neuropathy
6	52	Double blind
7	50	Orofacial pain
8	50	Clonazepam
9	49	Management
10	48	Alpha Lipoic acid

Prominent clusters include terms like “Pain modulation” (37), “Burning mouth syndrome” (34), “Symptoms” (32), “Dry mouth” (26),“Update” (24),“Clinical trials” (23),“Burning sensation” (22),“Nerve fibers” (22),“Iron”(19),“Allergy”(18),“Clonazepam” (16),“Saliva” (16),“Lichen planus” (15),“Gastric parietal cell antibody” (13) and “Neuropathic pain” (10) (Figure 11). Each cluster is color-coded to represent a distinct research theme or area, facilitating the identification of key trends and focal points within the research landscape. This analysis indicates that current research in BMS predominantly focuses on the management of chronic pain symptoms and the investigation of potential links between pathological indicators and etiology factors.

“Burst Keywords” are identified as terms that experience a significant increase in citations over a specific period, reflecting the emergence of research trends in a field. Figure 12 illustrates the top 20 keywords exhibiting the strongest citation bursts from 2008 to 2023. In this visual analysis, red bars represent terms that have gained significant scholarly attention and frequent citations during the specified period. Conversely, light green bars signify terms declining popularity or citation frequency, suggesting a decrease in scholarly focus or relevance during the respective periods. According to Figure 11, “management” emerges as the keyword with the longest burst duration of citation bursts, while terms like “blood profile,” “pernicious anemia” and “folate” may become the focal points of research in the forthcoming years.

## 4 Discussion

### 4.1 Overview of bibliometric findings

This bibliometric analysis reviewed 497 articles published between 2008 and 2023, revealing a consistent increase in publications related to BMS. The year 2022 marked a peak with 49 articles, the highest annual output since 2008. These articles were distributed across 180 different journals, with a majority in the field of oral sciences domain. The United States of America (USA) led in research contributions with 80 articles (16.0%), while the Netherlands and England were notable for their international collaborations. However, there is still a lack of BMS research institutions in regions like Africa, Southeast Asia and Greenland, possibly due to limited local attention to BMS. Factors such as dietary habits, healthcare standards and language barriers may also play a role in this disparity. Nihon University was identified as the leading institution in BMS research. The findings also indicate that most of the research efforts are concentrated in academic settings such as universities, while the broader clinical community may not fully recognize the importance of BMS.

Among individual contributors, Adamo Daniela was the most prolific, with 18 publications that delve into the relationship between BMS and psychological conditions such as anxiety and depression. Following closely was Sun Andy, who contributed 16 articles, highlighting their influential roles in the field. Adherence to both Price’s Law and Lotka’s Law confirmed the formation of a substantial core group of authors dedicated to BMS research.

The journal *Oral Diseases* published the highest number of articles on the topic, and visual overlay maps illustrated that BMS-related research often intersects with other medical disciplines such as dentistry, dermatology, and surgery. Co-citation analysis identified “Pathophysiology of primary burning mouth syndrome” as the most frequently co-cited article (24).

### 4.2 Current research trends and developments

A keyword analysis conducted using CiteSpace, for the period 2008 to 2023, identified “burning mouth syndrome” (358), “pain” (144) and “prevalence” (71) as the most prevalent terms. Further analysis, employing pathfinder analysis and the LLR algorithm, highlights “pain modulation” as a particularly prominent research theme.

“Pain modulation” emerges as a critical focus in the clinical investigation of chronic pain conditions (28–30). Key terms associated with this cluster include “pain modulation,” “temporal summation,” “functional MRI,” “fibromyalgia syndrome,” and “neuropathic pain.” Research exploring the central mechanisms of BMS has unveiled correlations between neuroprotective steroids and the modulation of emotional and pain responses within brain networks of patients (12). Studies incorporating somatosensory assessments, imaging, and electrophysiology reveal that BMS patients often demonstrate exaggerated pain responses and a dysregulation within the central nervous system’s pain modulation circuits (31). Furthermore, investigations into White Matter Hyperintensities (WMHs) in BMS patients indicate a correlation between a higher frequency of WMHs and increased pain perception, potentially leading to cognitive impairment and accelerated cerebral aging (32). WMHs are considered as early neuroimaging indicators of brain vulnerability (33–36). This implies that BMS research not only includes the study of chronic pain but also intersects with the exploration of neurodegenerative diseases. As such, ongoing research into BMS is poised to offer novel insights into chronic pain management and the pathophysiology of neurodegenerative disorders (8, 10, 37–40).

In the field related to BMS and conditions such as trigeminal neuralgia, the academic community is committed to optimizing surgical intervention strategies. These efforts aim to reduce complications and improve therapeutic outcomes (41). Within the broader context of neuropathic pain, there is a concerted effort to deepen the scientific understanding of such pain. This involves systematically evaluating treatment impacts, enhancing methods for prognostic assessments, introducing innovative treatment techniques, delving into the pathophysiological mechanisms, developing new diagnostic and screening tools, and assessing the multi-dimensional effects on the patients’ quality of life (42).

Specifically regarding BMS—a disease characterized by oral pain—research initiatives persistently explore its etiology and pathological process. In addition, there is a continuous focus on assessing the effectiveness of both single and combined treatment approaches. Research also examines the correlation between BMS symptoms and emotional disorders such as depression and anxiety. Among various aspects of neuropathic pain, the development of pain management strategies and the evaluation of treatment effects continue to be the focus of research areas.

According to the burst words analysis, “management” emerges as the keyword with the longest burst duration of increased frequency, indicating sustained interest in this area. Meanwhile, “blood profile,” “pernicious anemia” and “folate” are identified as likely focal points for future research. Observations indicate that BMS patients frequently exhibit poor health status, with micronutrients deficiencies playing a potential role in this condition (43). Notably, Sun Andy, a leading researcher in the field, has concentrated on examining the “blood profile” in BMS patients. His research has uncovered conditions like “pernicious anemia” and “folate deficiency” are prevalent among this patient group. Clinical investigations under his lead have assessed various indicators, including serum iron, Serum Ferritin (sFe), Folic Acid, Thyroid Globulin Antibodies (TGA), serum Gastric Parietal Cell Antibodies (GPCA) (44–47). Furthermore, his studies have demonstrated that abnormal serum homocysteine levels in BMS patients can be normalized through vitamin supplementation (48).

### 4.3 Strengths and limitations

This bibliometric study employs visual analytics to elucidate the current research landscape and emerging trends in BMS. However, this study primarily relied on manual literature screening, which might have introduced certain biases or omissions that could affect the accuracy of our findings. Additionally, the dependence on tools such as CiteSpace and VOSviewer limits the research to data available within the WoSCC. Expanding to additional databases would enhance the reliability of our conclusions and provide a more holistic overview. Future research would benefit from incorporating a broader range of databases to achieve more comprehensive analytical insights. In addition, our study highlights that ALA plays a key role in the treatment of BMS, as indicated by co-citation analyses (27). However, of the potential of more innovative therapies such as low-level laser (49) and emerging pharmacological treatments such as quetiapine (50) for BMS could not be explored in depth in this study, suggesting a gap in our understanding of future therapeutic directions.

## 5 Conclusion

Through bibliometric analysis, this study retrospectively examined 497 articles on BMS from 2008 to 2023. Our aim was to outline the current status of the field and forecast future research directions. The analysis indicates a growing academic interest in BMS and the establishment of a solid core of academic contributors. Despite the challenges of cross-agency and cross-regional cooperation, there is a trend towards more cohesive research approaches. Researchers from various countries and institutions might benefit from adopting integrated research methods, such as engaging in international cooperative research through multi-center interdisciplinary research. Given that chronic pain significantly impairs the quality of life and mental health of BMS patients, Research has increasingly focused on “pain modulation.” This emphasis aims to uncover more effective treatments by delving into the mechanisms of pain modulation, thereby improving the patients’ quality of life. Furthermore, emerging research avenues have highlighted the potential relevance of “blood profile,” “pernicious anemia” and “folate” to BMS pathogenesis, predicting their prominence in upcoming studies. A thorough exploration of these factors in relation to BMS is anticipated to foster innovative treatment paradigms and enrich the understanding of this complex condition.

## Author contributions

XL: Writing – review & editing, Writing – original draft. RJ: Writing – review & editing, Writing – original draft. WH: Writing – review & editing, Writing – original draft. YY: Writing – review & editing, Writing – original draft. JJ: Writing – review & editing, Writing – original draft. WZ: Writing – review & editing, Writing – original draft.
